# The impact of the tumor shrinkage by initial EGFR inhibitors according to the detection of *EGFR*-T790M mutation in patients with non-small cell lung cancer harboring EGFR mutations

**DOI:** 10.1186/s12885-018-5153-4

**Published:** 2018-12-11

**Authors:** Akihiro Yoshimura, Tadaaki Yamada, Naoko Okura, Takayuki Takeda, Wataru Furutani, Yutaka Kubota, Shinsuke Shiotsu, Osamu Hiranuma, Naoya Nishioka, Yusuke Chihara, Nobuyo Tamiya, Yoshiko Kaneko, Junji Uchino, Koichi Takayama

**Affiliations:** 1 Department of Pulmonary Medicine, Graduate School of Medical Science, Kyoto Prefectural University of Medicine, 465, Kajii-cho, Kamigyo-ku, Kyoto, 602–8566 Japan; 2 Department of Respiratory Medicine, Uji-Tokushukai Medical Center, Uji, Japan; 3 Department of Respiratory Medicine, Japanese Red Cross Kyoto Daini Hospital, Kyoto, Japan; 4 Department of Respiratory Medicine, Japanese Red Cross Kyoto Daiichi Hospital, Kyoto, Japan; 5 Department of Respiratory Medicine, Otsu city hospital, Otsu, Japan

**Keywords:** EGFR-T790M mutation, Non-small cell lung cancer, Re-biopsy, Tumor shrinkage

## Abstract

**Background:**

The *EGFR*-T790M mutation is clinically detected using re-biopsy in approximately 50% of patients with acquired resistance to epidermal growth factor receptor tyrosine kinase inhibitors (EGFR-TKIs) in advanced non-small cell lung cancer (NSCLC) who harbor EGFR mutations. However, little is known about the population of NSCLC patients who develop acquired resistance due to the T790M mutation. In this study, we focused on the emergence of the T790M mutation and analyzed patients refractory to initial EGFR-TKIs with successful re-biopsy samples.

**Methods:**

Seventy-eight advanced NSCLC patients with EGFR mutations who had successful re-biopsy samples after resistance to initial EGFR-TKI treatment were enrolled at 5 institutions in Japan. We validated the association between the emergence of the T790M mutation and their clinical profiles.

**Results:**

Thirty-nine patients tested positive for T790M and 39 tested negative in the re-biopsy samples. The objective response rate to initial EGFR-TKIs was higher in patients with the T790M mutation than in those without the mutation (89.7% versus 51.2%, *p* < 0.001). Moreover, there was a significant difference in the maximal tumor shrinkage rate relative to baseline in T790M-positive tumors compared with T790M-negative tumors (42.7% versus 24.0%, *p* = 0.001). Multivariate analysis demonstrated that the maximum tumor shrinkage rate was a significant predictive factor for the detection of the T790M mutation (*p* = 0.023, odds ratio 1.03, 95% confidence interval 1.00–1.05).

**Conclusions:**

Our retrospective observations suggested that the maximum tumor shrinkage rate with initial EGFR-TKI treatment might be one of the promising predictive biomarkers for emerging refractory tumors with the *EGFR*-T790M mutation.

**Electronic supplementary material:**

The online version of this article (10.1186/s12885-018-5153-4) contains supplementary material, which is available to authorized users.

## Background

Non-small cell lung cancer (NSCLC) with activating epidermal growth factor receptor (EGFR) mutations, such as exon 19 deletion and the L858R mutation, responds to first and second generation EGFR-tyrosine kinase inhibitors (EGFR-TKIs) [1, 2]. However, it ultimately acquires resistance to EGFR-TKIs after various periods of time. Acquired resistance to initial EGFR-TKIs is caused by various mechanisms, such as gatekeeper mutations like the *EGFR*-T790M second site mutation, activation of bypass signaling, and transformation to small-cell lung cancer. *EGFR*-T790M, the gatekeeper mutation, is the most common mechanism of acquired resistance and is detectable in approximately 50% of patients who experience acquired resistance to first and second generation EGFR-TKIs [3].

Osimertinib is a third generation EGFR-TKI that inhibits EGFR with activating mutations and/or the T790 M resistance mutation. Osimertinib has been approved in several countries, including the U.S.A and Japan, for the treatment of *EGFR*-T790M-positive NSCLC patients with tumors that are refractory to first or second generation EGFR-TKIs [4]. In addition, osimertinib has been approved in several countries for the first-line treatment of patients with *EGFR*-mutated NSCLC, based on the results of the FLAURA study [5].

We usually detect the *EGFR*-T790M mutation using re-biopsy of tissue, although the procedure is relatively invasive and is not always feasible to perform. Recently, liquid biopsy has received much attention as a non-invasive method to detect *EGFR*-T790M mutations. However, tissue re-biopsy may still be needed if the liquid biopsy is negative or if other mechanisms of resistance, such as transformation to small cell histology, are being probed. Therefore, elucidation of the clinical profiles underlying the emergence of acquired resistance to initial EGFR-TKIs with the T790M mutation has led us to perform the re-biopsy approach initially.

The goal of this research is to identify a promising clinical biomarker for the detection of the *EGFR*-T790M mutation using re-biopsy in EGFR-mutated NSCLC patients, based on patient profiles.

## Methods

### Patients

We retrospectively enrolled 78 advanced or relapsed NSCLC patients with active EGFR mutations without *EGFR*-T790M. All patients had re-biopsy samples originating from either tumors or plasma after developing acquired resistance to initial EGFR-TKIs at 5 institutions in Japan between May 2014 and January 2018 (Table 1).

**Table 1:** Patient characteristics according to EGFR-T790M mutation status

Characteristic	T790M + (*n* = 39)	T790M - (*n* = 39)	*p* value
	n (%)	n (%)	
Age			
Median (Range)	73.0 (44.0–85.0)	70.0 (55.0–88.0)	0.224
Sex			
Male	13 (33.3)	15 (38.5)	0.814
Female	26 (66.7)	24 (61.5)	
PS			
0	26 (66.7)	23 (59.0)	0.682
1	11 (28.2)	12 (30.8)	
2	2 (5.1)	4 (10.2)	
Histology			
Adenocarcinoma	38 (97.4)	33 (84.6)	0.025
Squamous cell carcinoma	0 (0.0)	6 (15.4)	
NSCLC	1 (2.6)	0 (0.0)	
Smoking status			
Never-smoker	26 (66.6)	23 (59.0)	0.72
Ever-smoker	6 (15.4)	7 (17.9)	
Current-smoker	6 (15.4)	9 (23.1)	
NE	1 (2.6)	0 (0.0)	
Stage			
III	5 (12.8)	3 (7.7)	0.267
IV	21 (53.8)	28 (71.8)	
Postoperative recurrence	13 (33.4)	8 (20.5)	
EGFR mutation status			
Exon 19 deletion	26 (66.7)	20 (51.3)	0.25
Exon 21 L858R	13 (33.3)	18 (46.1)	
Other	0 (0.0)	1 (2.6)	
EGFR-TKI			
Gefitinib	23 (59.0)	29 (74.4)	0.369
Erlotinib	9 (23.1)	5 (12.8)	
Afatinib	7 (17.9)	5 (12.8)	
ORR			
CR/PR	35 (89.7)	20 (51.3)	< 0.001
SD	4 (10.3)	18 (46.1)	
PD	0 (0.0)	1 (2.6)	
Re-biopsy site			
within thorax	11 (28.2)	13 (33.3)	0.77
out of thorax	22 (56.4)	22 (56.4)	
plasma	6 (15.4)	4 (10.3)	
MTS (%)			
Median (Range)	42.7 (8.0–100.0)	24.0 (−17–100.0)	0.001
PFS duration			
< 6 months	3 (7.7)	10 (25.6)	0.065
> =6 months	36 (92.3)	29 (74.4)	

All patients underwent image evaluation using a conventional computed tomography (CT) scan according to Response Evaluation Criteria in Solid Tumors (RECIST) version 1.1. The maximal tumor shrinkage (MTS) was defined as the highest tumor shrinkage rate relative to baseline according to the CT image during treatment with initial EGFR-TKIs. We obtained the following clinical data from retrospective medical records; age, sex, histological subtype, EGFR mutation status, disease stage, Eastern Cooperative Oncology Group Performance Status (PS), smoking status, progression-free survival (PFS), objective response rate (ORR), and MTS of patients on initial EGFR-TKIs. The study protocol was approved by the Ethics Committees of each hospital. The TNM stage was classified using version 7 of the TNM stage classification system.

### Genomic analysis

EGFR mutations were detected using polymerase chain reaction for tumor and plasma samples with sequencing of exons 18–21 performed at commercial clinical laboratories; SRL, Inc. and BML, Inc. (Tokyo, Japan).

### Statistical analysis

Cox proportional hazards models considering several patient factors were used. The cut-off point in MTS was determined using receiver operating characteristic (ROC) curve analysis. To analyze PFS, times to events were estimated using the Kaplan-Meier method and compared using the log-rank test. PFS was censored at the date of disease progression. Predictive factors for the detection of *EGFR*-T790M were identified using univariate and multivariate logistic analyses. All statistical analyses were performed using SPSS 25.0 for Windows (SPSS Inc., Chicago, IL, USA). All *p* values less than 0.05 were considered statistically significant.

## Results

### Patient characteristics

Thirty-nine patients had *EGFR*-T790M-positive disease and 39 had negative disease in the re-biopsy samples. Thirteen (33.3%) and 15 (38.5%) patients were male, 26 (66.6%) and 23 (59.0%) were never smokers, and the median age was 73.0 years (range, 44–85 years) and 70.0 years (range, 55–88 years) in the *EGFR*-T790M positive and negative groups, respectively. The histological subtypes were adenocarcinoma in 38 (97.4%) and NSCLC in 1 (2.6%) patient with T790M-positive disease, and adenocarcinoma in 33 (84.6%) and squamous cell carcinoma in 6 (15.4%) patients with T790M negative disease, respectively. In terms of *EGFR* mutations at baseline, 26 (66.7%) and 20 (51.3%) patients harbored a deletion in exon 19, while 13 (33.3%) and 18 (46.1%) patients had a L858R missense mutation in exon 21 in the T790M positive and negative groups, respectively (Table 1).

### Association between clinical features and the emergence of EGFR-T790M mutation detected using re-biopsy samples in EGFR-mutant NSCLC patients

Among patient profiles, the histological subtype significantly correlated with the emergence of the *EGFR*-T790M mutation (*p* = 0.025) (Table 1). Thirty-five (89.7%) patents with our observations showed that re-biopsy tissue samples came from within the thorax in 44 patients and from outside of the thorax in 24 patients. There was no significant correlation among re-biopsy sites for the detection of the *EGFR*-T790M mutation (within the thorax, outside of the thorax, and plasma; 28.2, 56.4 and 15.4%, respectively, *p* = 0.770). The detection rate of the T790M mutation in tissue and plasma was 48.5 and 60.0%, respectively. There was no significant difference in the re-biopsy method for the detection of *EGFR*-T790M (*p* = 0.737).

**Fig. 1:**
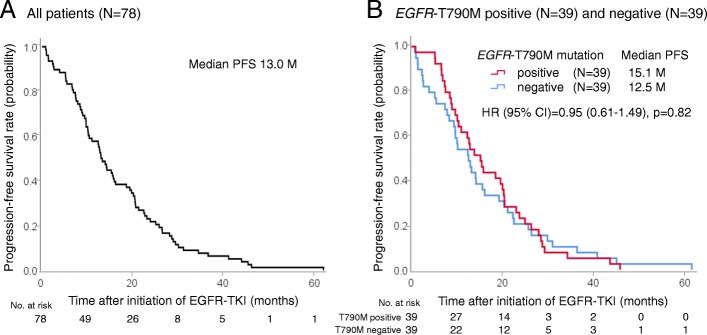
Kaplan-Meier survival curves for PFS. **a** PFS of all patients (*N* = 78) on initial EGFR-TKI treatment. **b** PFS of patients with *EGFR*-T790M mutation (*N* = 39) or without *EGFR*-T790M mutation (*N* = 39) on initial EGFR-TKI treatment

**Fig. 2:**
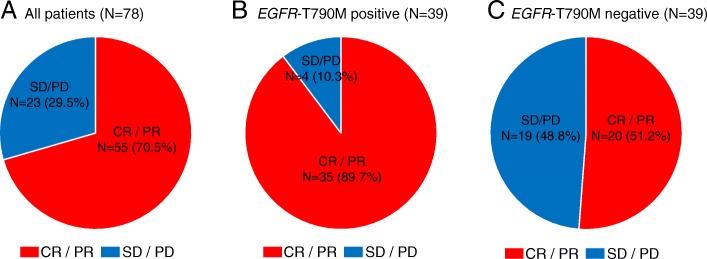
Frequency of best overall response to initial EGFR-TKIs in EGFR mutated NSCLC. **a** Frequency of best overall response to initial EGFR-TKI treatment among all patients (*N* = 78). **b** Frequency of best overall response to initial EGFR-TKI treatment among all patients with the *EGFR*-T790M mutation (*N* = 39). **c** Frequency of best overall response to initial EGFR-TKI treatment across all patients without the *EGFR*-T790M mutation (*N* = 39)

**Fig. 3:**
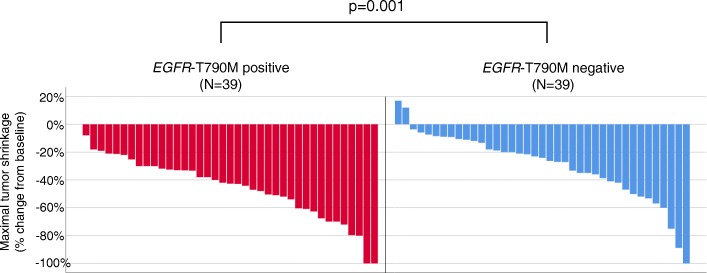
Waterfall plot of patients with EGFR-mutated NSCLC treated with initial EGFR-TKIs. The average tumor shrinkage rate relative to baseline in 78 patients with EGFR-mutated NSCLC treated with initial EGFR-TKIs. Re-biopsy samples obtained after the development of acquired resistance to initial EGFR-TKIs revealed that 39 patients had the T790M mutation (red bar) and 39 patients did not have the T790M mutation (blue bar). The median tumor shrinkage rate in these patients indicated a significant association with the emergence of *EGFR*-T790M (42.7 and 24.0%, respectively, *p* = 0.001)

Median PFS with initial EGFR-TKI was 13.0 months in all NSCLC patients. There was no significant correlation in PFS of initial EGFR-TKI in *EGFR*-T790M positive patients compared with *EGFR*-T790M negative patients (15.1 months and 12.5 months, respectively, *p* = 0.82) (Fig. 1). The ORR was higher in patients with T790M-positive disease than in those with T790M-negative disease (89.7% versus 51.2%, *p* < 0.001) (Fig. 2). Given that the ORR to EGFR-TKIs was closely correlated with the emergence of *EGFR*-T790M-positive disease, we next examined the MTS rate relative to baseline according to exposure to initial EGFR-TKIs. The median MTS rate of *EGFR*-T790M-positive and -negative NSCLC was 42.7% (range, 8.0–100.0%) and 24.0% (range, −17–100.0%), respectively, which indicated a significant association with the emergence of *EGFR*-T790M (*p* = 0.001) (Fig. 3). ROC curve analysis demonstrated that the optimal MTS cutoff for the emergence of the *EGFR*-T790M mutation was 30% (82.1% sensitivity and 59.0% specificity, area under the curve 0.713, *p* < 0.001) (Additional file 1: Figure S1). Thirty-five (63.6%) patients with MTS rate more than 30% had an *EGFR*-T790M mutation, while 4 (17.4%) patients with MTS rate less than 30% had an *EGFR*-T790M mutation. Finally, multivariate analysis demonstrated that MTS was a significant predictive factor for the detection of *EGFR*-T790M using re-biopsy (odds ratio, 1.03; 95% confidence interval, 1.00–1.05; *p* = 0.023) (Table 2).

**Table 2:** Predictive factors for the emergence of EGFR-T790M mutation according to multivariate analysis

Variables	Odds ratio	*p* value
Histology		
Adenocarcinoma (vs. other)	0.35 (0.06–2.05)	0.24
EGFR mutation status		
Exon 19 deletion (vs. other)	0.52 (0.20–1.37)	0.18
MTS		
The levels of shrinkage	1.03 (1.00–1.05)	0.023
PFS duration		
> =6 months (vs. < 6 months)	2.43 (0.54–11.00)	0.25

## Discussion

Clinically, it is critical to select the population with the *EGFR*-T790M mutation when we consider obtaining re-biopsy samples from tumors and/or blood to decide on treatment with osimertinib. Previous reports have described various potential factors regarding the emergence of the *EGFR*-T790M mutation, such as the duration of initial EGFR-TKI treatment, *EGFR* gene status at baseline, and surgical history [6–8]. Our findings were inconsistent with these factors as predictors of the emergence of the T790 M mutation, while these other predictive factors have showed controversial results for the detection of the T790M mutation [6]. Therefore, a novel powerful biomarker is needed to predict the emergence of the T790M mutation using re-biopsy samples.

Although our observations showed that the ORR to EGFR-TKIs is one of the significant predictive biomarkers, it will be difficult to closely evaluate the response of EGFR-TKI using RECIST categorized into only 4 groups: complete response (CR), partial response (PR), stable disease (SD), and progression disease (PD). To further assess for the response to EGFR-TKIs, we validated the MTS rate as a novel evaluation method. A previous report showed that the MTS rate was not a good prognostic factor in EGFR mutation-positive NSCLC [9]. We have clearly shown here that the increased MTS rate in response to initial EGFR-TKIs was significantly associated with the emergence of the *EGFR*-T790M mutation among patients with NSCLC tumors with acquired resistance. Further, the optimal cutoff for the MTS rate in relation to the emergency of the *EGFR*-T790M mutation was 30%, which is the same as the RECIST-PR level. Crucially, multivariate analysis indicated that the MTS rate was an independent predictive factor for the emergence of the T790M mutation using re-biopsy; this showed a greater association than the duration of initial EGFR-TKI treatment, *EGFR* gene status, and tumor histology, as previously described.

Tumors with the *EGFR*-T790M mutation showed a relatively low tumor mutation burden (TMB) in next generation sequencing analysis [10], whereas tumors without the *EGFR*-T790 M mutation with EGFR-TKI acquired resistance showed molecular heterogeneity [11]. In fact, TMB was reported to be a promising predictor of the detection of the *EGFR*-T790M mutation [12]. These observations suggest that the original de novo tumors that ultimately acquire resistance with the T790M mutation are more likely to retain EGFR signal directivity than those without the T790M mutation. Similarly, our retrospective findings showed that de novo EGFR mutated tumors with good responses to initial EGFR-TKIs have a higher dependency on EGFR signaling in cases of acquired T790M mutation than those without the T790M mutation, suggesting that the MTS rate with initial EGFR-TKIs may be a useful predictor of the emergence of the T790M mutation using re-biopsy.

This study has several limitations. Firstly, it comprised a small retrospective sample. However, previous retrospective observations have used similar sample sizes, with occasional exceptions [6–8]. Second, we only considered a Japanese cohort. Third, there may have been bias considering when EGFR-TKI was started, even though treatment was administered in multiple centers, and in the timing of evaluation using CT scanning, even though it was performed every 1–3 months after treatment. Therefore, a further prospective study is warranted to identify the role of the MTS rate for detecting the emergence of the *EGFR*-T790 mutation in NSCLC following initial EGFR-TKI treatment.

## Conclusions

Our retrospective observations suggested that the MTS rate in response to initial EGFR-TKI treatment might be one of the promising predictors for the emergence of refractory tumors with the EGFR-T790M mutation. Further experiments are needed to validate this.

## Additional file

Additional file 1: Receiver operating characteristics curve analysis for the optimal cutoff of the most tumor shrinkage rate relative to baseline. (PPTX 53 kb)

## Abbreviations

CR: Complete response
CT: Computed tomography
EGFR: Epidermal growth factor receptor
MTS: Maximal tumor shrinkage
NSCLC: Non-small cell lung cancer
ORR: Objective response rate
PD: Progressive disease
PFS: Progression-free survival
PR: Partial response
PS: Eastern Cooperative Oncology Group Performance Status
RECIST: Response Evaluation Criteria in Solid Tumors
ROC: Receiver operating characteristic
SD: Stable disease
TKI: Tyrosine kinase inhibitor
TMB: Tumor mutation burden
