# Supplementary material for: The impact of the tumor shrinkage by initial EGFR inhibitors according to the detection of EGFR-T790M mutation in patients with non-small cell lung cancer harboring EGFR mutations
Source: BMC Cancer. 2018 Dec 11;18:1241. doi: 10.1186/s12885-018-5153-4 (PMC6288862; doi:10.1186/s12885-018-5153-4)
Supplement: Supplementary file 1 — Receiver operating characteristics curve analysis for the optimal cutoff of the most tumor shrinkage rate relative to baseline. (PPTX 53 kb) [file 12885_2018_5153_MOESM1_ESM.pptx]

## Slide 1
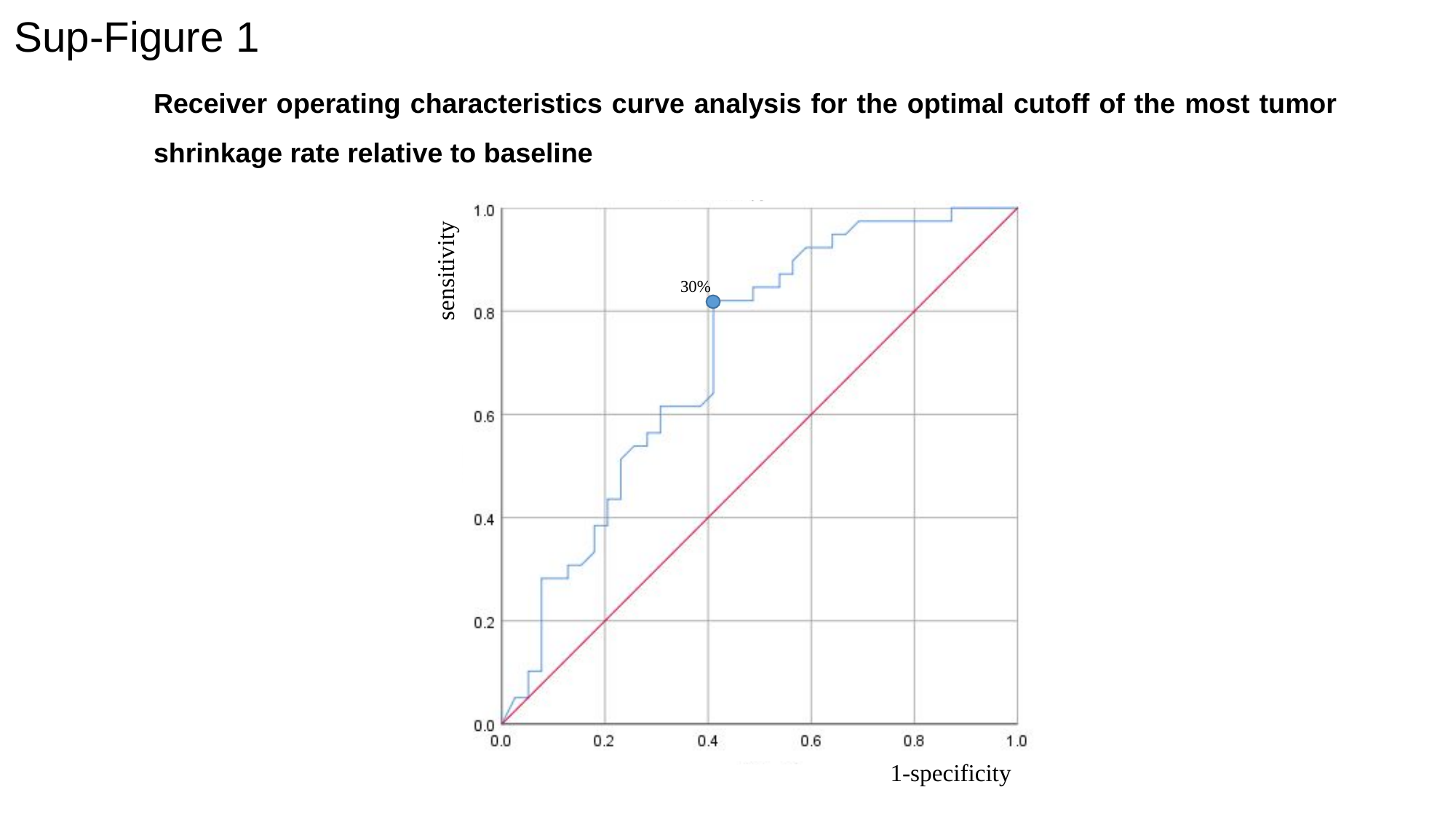

Sup-Figure 1
Receiver operating characteristics curve analysis for the optimal cutoff of the most tumor shrinkage rate relative to baseline
sensitivity
30%
1-specificity
